# Search for efficient inhibitors of myotoxic activity induced by ophidian phospholipase A_2_-like proteins using functional, structural and bioinformatics approaches

**DOI:** 10.1038/s41598-018-36839-6

**Published:** 2019-01-24

**Authors:** Guilherme H. M. Salvador, Fábio Florença Cardoso, Antoniel A. Gomes, Walter L. G. Cavalcante, Márcia Gallacci, Marcos R. M. Fontes

**Affiliations:** 1 Depto. de Física e Biofísica, Instituto de Biociências, UNESP – Universidade Estadual Paulista, Botucatu, SP Brazil; 2 Depto. de Farmacologia, UFMG - Universidade Federal de Minas Gerais, Belo Horizonte, MG Brazil; 3 Depto. de Farmacologia, Instituto de Biociências, UNESP – Universidade Estadual Paulista, Botucatu, SP Brazil

## Abstract

Ophidian accidents are considered an important neglected tropical disease by the World Health Organization. Particularly in Latin America, *Bothrops* snakes are responsible for the majority of the snakebite envenomings that are not efficiently treated by conventional serum therapy. Thus, the search for simple and efficient inhibitors to complement this therapy is a promising research area, and a combination of functional and structural assays have been used to test candidate ligands against specific ophidian venom compounds. Herein, we tested a commercial drug (acetylsalicylic acid, ASA) and a plant compound with antiophidian properties (rosmarinic acid, RA) using myographic, crystallographic and bioinformatics experiments with a phospholipase A_2_-like toxin, MjTX-II. MjTX-II/RA and MjTX-II/ASA crystal structures were solved at high resolution and revealed the presence of ligands bound to different regions of the toxin. However, *in vitro* myographic assays showed that only RA is able to prevent the myotoxic effects of MjTX-II. In agreement with functional results, molecular dynamics simulations showed that the RA molecule remains tightly bound to the toxin throughout the calculations, whereas ASA molecules tend to dissociate. This approach aids the design of effective inhibitors of PLA_2_-like toxins and, eventually, may complement serum therapy.

## Introduction

Snakebite accidents caused by *Bothrops* snakes can induce severe local myonecrosis in victims, which may lead to permanent sequelae, such as drastic tissue loss, amputation and disability^1–4^. Serum therapy is the most used treatment for this pathology; however, the difficulty of neutralizing the local effect is a challenge, since the rapid action of toxins present in *Bothrops* venom reduces the serum efficiency^1,5,6^.

One class of proteins present in bothropic venom is the phospholipases A_2_ (PLA_2_s), which can be divided in catalytic PLA_2_s and myotoxic PLA_2_-like proteins^7–9^. Several sites responsible for myotoxic activity caused by myotoxic PLA_2_-like proteins (including Lys49-PLA_2_s) were previously described and led to the current proposed myotoxic mechanism^6,10–13^. A step-by-step myotoxic mechanism for PLA_2_-like proteins was presented initially in 2014 and complemented in 2017; the mechanism consists of several steps, including: *i)* hydrophobic molecule binding at the hydrophobic channel; *ii)* protein activation - including protein reorientation and stabilization with exposure of its active sites to the solvent; *iii)* protein-membrane interaction at the membrane-docking site (MDoS); *iv)* membrane destabilization by residues at the membrane-disrupting site (MDiS); and *v)* cell death^14,15^.

Interestingly, myotoxin-II (MjTX-II) isolated from *Bothrops moojeni* venom presents some structural differences compared to other PLA_2_-like proteins from the *Bothrops* genus^16,17^. The changes in its amino acid sequence, including mutations in conserved residues (Leu32Gly and His121Tyr) and the insertion of an Asn residue at position 120, are reflected in structural changes in the hydrophobic channel and its interactions with long-chain molecules^16–18^. Furthermore, fatty acids binding to MjTX-II are not necessary for the alignment of its MDoS and MDiS regions^18^; consequently, this difference is important compared to other PLA_2_-like toxins, since MjTX-II activation would be facilitated.

Snakebite envenomation is considered a neglected tropical disease by the World Health Organization. It leads to approximately one 100,000 deaths per year and around three times as many amputations and other permanent disabilities due to poor access to a health system and the inefficiency or lack of specific antivenoms. Thus, the search for simple and efficient inhibitors to complement serum therapy is a promising research area. In the last decade, a combination of functional, biochemical, biophysical, structural and bioinformatics tools have been used to test ligands that may be candidates to specifically inhibit ophidian venom compounds^17,19–25^.

Medicinal plants, which are used in folk medicine, are a source of biological active compounds, including inhibitor candidates for the snake envenoming^26–30^. Plants from *Boraginaceae* and *Laminaceae* families have several important biological properties, including its ability to neutralize the inflammatory, myotoxic and hemorrhagic activities of crude snake venoms and/or their isolated toxins^21,31^. Rosmarinic acid (RA) is a polyphenolic compound found in those plants^21,32^, and more recently, several studies have demonstrated the efficiency of this molecule against the myotoxic effects induced by different *Bothrops* venoms and isolated toxins^20,21,31,33,34^. By contrast, acetylsalicylic acid (ASA) is a largely used commercial drug with anti-inflammatory and analgesic activities^35,36^. ASA inhibited the catalytic activity of pancreatic PLA_2_^37^ but was not tested against the myotoxic effects induced by PLA_2_-like proteins from snake venom.

Thus, in the present work, three different techniques (myography, crystallography and bioinformatics assays) were used to test the potential inhibitory characteristics of two different ligands, acetylsalicylic acid (ASA) and rosmarinic acid (RA), against the PLA_2_-like myotoxin MjTX-II. In addition, these functional and structural experiments were used to obtain additional insight into the myotoxic mechanism of MjTX-II (and consequently of other PLA_2_-like myotoxins) and in the search for efficient inhibitors of PLA_2_-like proteins.

## Results

### Neuromuscular blocking activity

**Figure 1:**
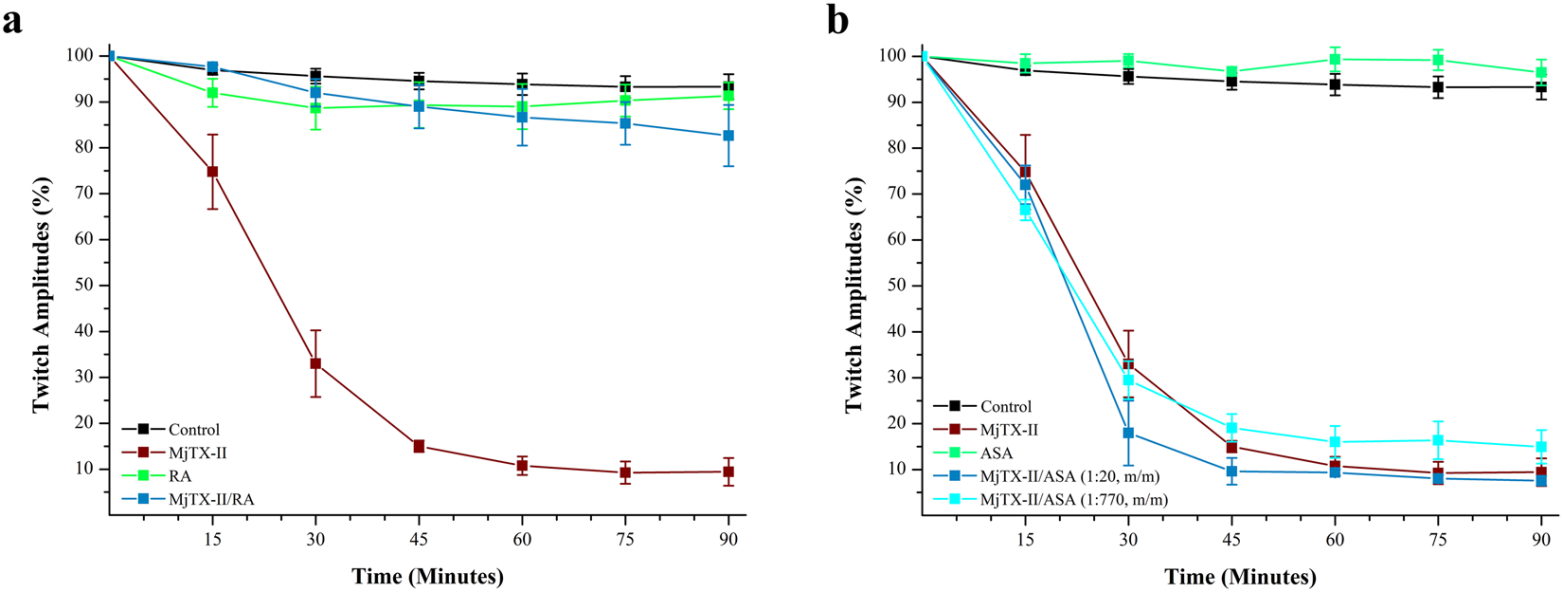
Effects of the MjTX-II and the product of its pre-incubation with (**a**) rosmarinic acid (RA) and (**b**) acetylsalicylic acid (ASA) on indirectly evoked twitches in isolated mice phrenic diaphragm preparations. The ordinate indicates the percentage of twitches relative to its initial amplitude. The abscissa indicates the time (minutes) after addition of MjTX-II, (**a**) rosmarinic acid or (**b**) acetylsalicylic acid alone and the mixture of MjTX-II plus (**a**) rosmarinic acid (1:10, m/m) or (**b**) acetylsalicylic acid (1:20, m/m and 1:770, m/m) to the organ bath. The data are grouped as means ± SEM (*P* < 0.05) and all groups have *N* = 3. *Indicates the point from which there was a significant difference compared with control.

*In vitro* myographic assays of the indirectly evoked contractions in nerve-muscle preparations has been a very sensitive model to functionally evaluate the myotoxic activity of PLA_2_-like toxins^17–20,23,38–41^. MjTX-II (1 μM, *N* = 3) promoted a time-dependent blockage of indirectly evoked twitches in mouse phrenic-diaphragm (PD) preparations. After 90 minutes, the twitch amplitudes were reduced by 92.6% (Fig. 1). The paralyzing effect of MjTX-II could not be reversed by washing the preparation for at least 30 minutes with toxin-free Ringer’s physiological solution (data not shown). RA prevented approximately 87.3% of the muscle paralysis promoted by MjTX-II in 90 minutes when both were pre-incubated (at a toxin:inhibitor ratio of 1:1, w/w., *i.e.*, 1:40, m/m; *N* = 3) (Fig. 1a). However, ASA did not prevent the neuromuscular blockage in 90 minutes when MjTX-II and this compound were pre-incubated (at a toxin:drug ratio of 1:20, m/m; *N* = 3) (Fig. 1b). Alone, RA (40 μM, *N* = 3) and ASA (20 μM, *N* = 3) did not affect the muscle contractions (Fig. 1).

### Crystallographic structures

The crystal structures of both MjTX-II complexes present similar folding found in class-II PLA_2_s solved to date, including seven disulfide bridges in each protomer and the following structural features: an N-terminal α-helix; a “short” helix; a Ca^2+^ binding loop (non-functional for PLA_2_-like toxins); two α-helices; two short strands of anti-parallel β-sheet (β-wing) and a C-terminal loop^10,42^.

**Figure 2:**
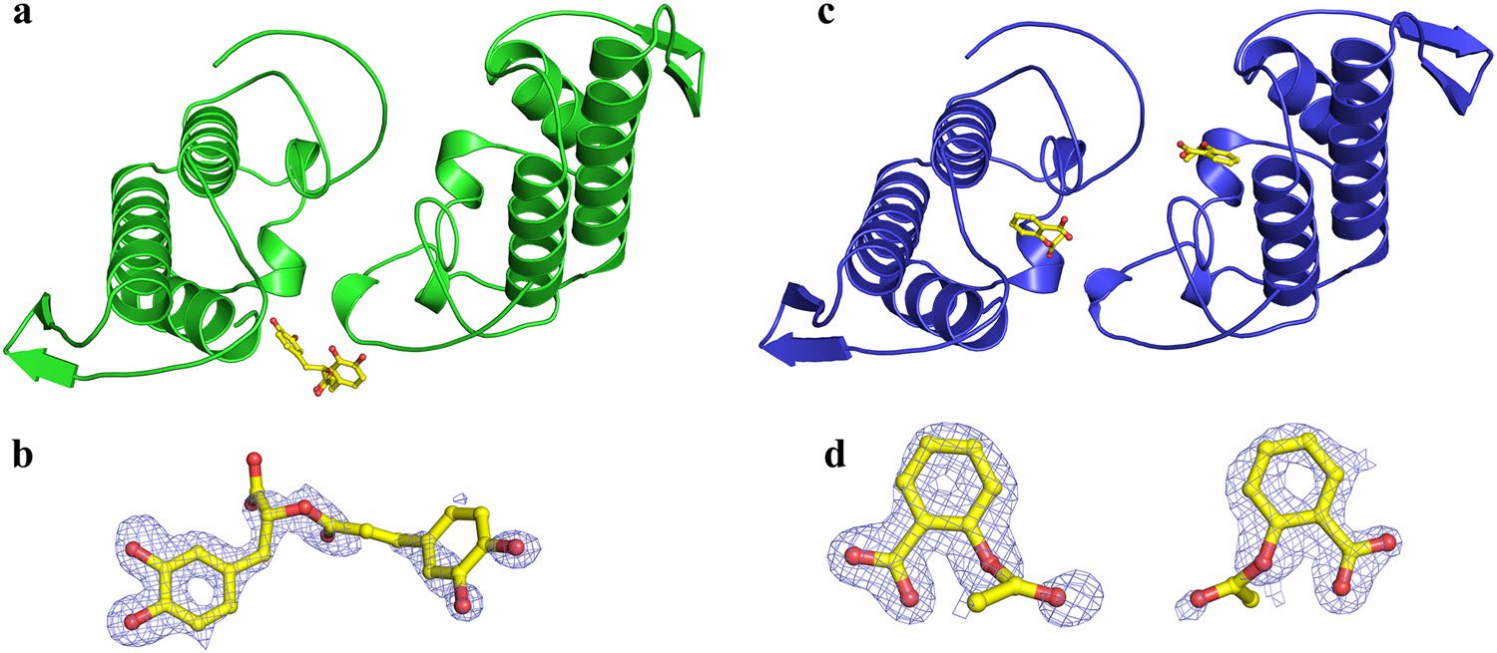
Crystal structures of MjTX-II/rosmarinic acid (RA) and MjTX-II/ acetylsalicylic acid (ASA) complexes. (**a**) The overall structure of the MjTX-II/RA complex is shown as a cartoon representation. The RA (yellow) molecule is illustrated as stick representation. (**b**) Omit electron density map (coefficients 2|F_obs_| - |F_calc_|) corresponding to RA is contoured at 1.0σ. (**c**) The overall structure of the MjTX-II/ASA complex is shown as a cartoon representation. ASA (yellow) molecules are illustrated as stick representation. (**d**) Omit electron density map (coefficients 2|F_obs_| - |F_calc_|) corresponding to the ASA molecules bound to monomer A and B, respectively, are contoured at 1.0σ.

The MjTX-II/RA crystal diffracted up to 1.60 Å and belonged to the P2_1_2_1_2_1_ space group. The refinement converged to an R_cryst_ of 15.7% (R_free_ = 19.1%) with one RA molecule located close to the MDiS and helix 1 from monomer A and 392 water molecules (Table 1, Fig. 2a and b). Due to the lack of electron density, the side chains of the following residues were not modeled: Lys69 and Lys116 from monomer A and Lys70, Lys115, Lys128 and Lys129 from monomer B.

**Table 1:** X-ray data collection and refinement statistics for MjTX-II/RA and MjTX-II/ASA structures.

	MjTX-II/RA	MjTX-II/ASA
Space Group	P2_1_2_1_2_1_	P2_1_
Unit Cell (Å, °)	a = 46.1; b = 65.8; c = 90.4	a = 39.6; b = 65.6; c = 55.1; β = 92.7
Resolution range (Å)	23.55–1.59 (1.65–1.59)	18.32–1.69 (1.75–1.69)
Unique reflections	37011 (3613)	29552 (2767)
Multiplicity	9.7 (4.7)	3.3 (3.2)
Completeness (%)	99.7 (98.7)	94.0 (88.9)
Mean *I/σ* (*I*)	21.82 (3.0)	16.3 (2.4)
Molecules in ASU	2	2
Matthews coefficient V_M_ (Å^3^Da^−1^)	2.5	2.5
*R*_*merge*_^b^ (%)	10.5 (72.3)	4.9 (53.3)
Reflections used in refinement	37010 (3613)	29545 (2764)
Reflections used for R_free_	1851 (181)	1441 (129)
*R*_*work*_	15.8 (20.7)	17.9 (23.4)
*R*_*free*_	19.0 (23.7)	21.8 (28.7)
Number of non-hydrogen atoms		
Protein	1913	1908
Water	392	314
Ligands	26	38
Rosmarinic acid / CC	1/0.8	—
Acetylsalicylic acid / CC	—	2/0.8; 0.8
Average B-factor		
Overall	21.3	25.2
Protein	18.9	23.6
Ligands	35.4	39.1
Solvent	31.9	33.8
Ramachandran Plot (%)^c^		
Favored	97.1	96.7
Outliers	0	0
Rotamer outliers (%)	1.4	0
C_β_ outliers	0	0
Clash score	3.17	6.84
RMS (bonds) (Å)	0.013	0.013
RMS (angles) (°)	1.57	1.16

Numbers in parenthesis are for the highest resolution shell.

The MjTX-II/ASA crystal diffracted up to 1.69 Å resolution and belonged to the P2_1_ space group. The final refined data converged to an R_cryst_ of 18.1% (R_free_ = 21.8%), with two ASA molecules interacting with the hydrophobic channel from each monomer, 3 DMSO molecules, and 314 water molecules (Table 1, Fig. 2c,d). Due to the lack of electron density, the side chains of Lys7, Lys60 and Lys117 residues from monomer A and Lys53, Lys69, Lys105 and Lys117 residues from monomer B are not modeled. Both crystal structures (coordinates and structure factors) were deposited into the Protein Data Bank (PDB) under the following ids: 6MQD (MjTX-II/RA) and 6MQF (MjTX-II/ASA).

**Figure 3:**
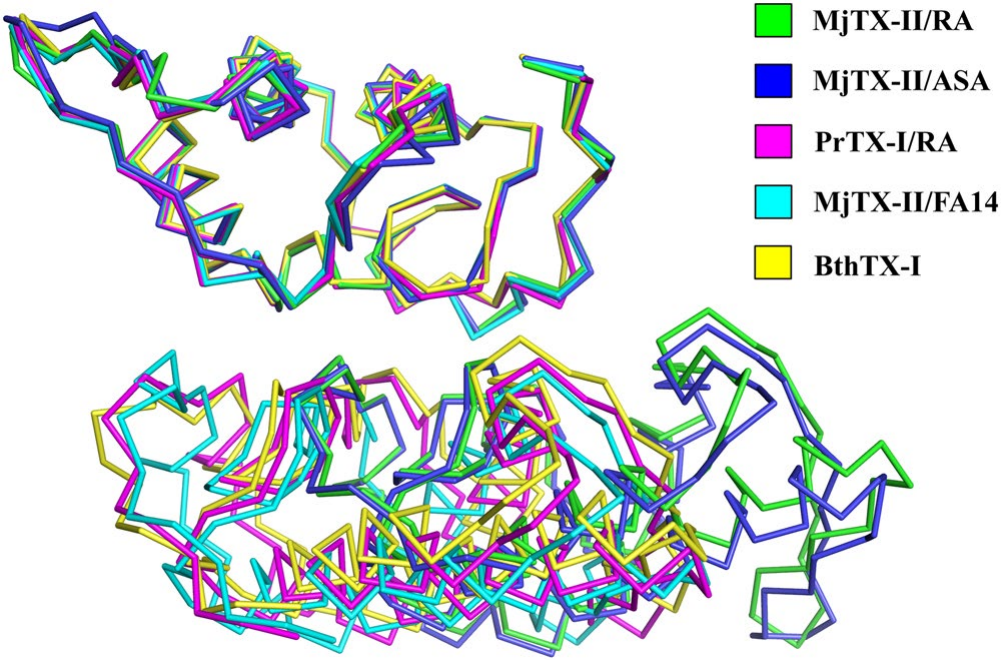
Superposition of C_α_ atoms of one monomer from each of PLA_2_s-like structures: MjTX-II/RA (green), MjTX-II/ASA (blue), PrTX-I/RA (magenta), MjTX-II/FA14 (cyan) and BthTX-I (yellow) and the relative disposition of other monomers of each structure.

The superposition between the PLA_2_-like myotoxins (*e*.*g*., PrTX-I/RA, MjTX-II/FA14 and BthTX-I) and the structures solved here revealed that the MjTX-II/RA and MjTX-II/ASA structures present a distorted dimeric assembly (Fig. 3) as expressed by Euler angles^15^ and center of mass displacement (COMdisp) values (see Methods section) (Table 2).

**Table 2:** Calculated angles^15^, center of mass displacement (COMdisp) and radius of gyration (R_g_) for MjTX-II/RA, MjTX-II/ASA and other PLA_2_-like toxins quaternary structures.

PDB id	Toxin	Roll (°)	Twist (°)	Tilt (°)	COMdisp (Å)	R_g_ (Å)
3IQ3	BthTX-I/PEG4K	178	28	27	2.5	18.1
4K06	MTX-II/PEG4K	178	28	27	3.0	18.3
3QNL	PrTX-I/RA	173	24	34	1.7	18.5
6B84	*apo*-MjTX-II	164	22	73	7.9	18.7
6B81	MjTX-II/FA8	167	24	59	4.0	18.5
6B80	MjTX-II/FA14	170	21	51	0.0	18.5
6MQF	MjTX-II/ASA*	165	36	41	12.7	20.1
6MQD	MjTX-II/RA	166	35	47	13.3	20.0
-^#^	MjTX-II/RA	198	62	67	31.5	21.8
-^#^	MjTX-II/ASA	130	7	38	3.3	20.1
-^#^	Unbound MjTX-II	167	6	12	9.7	20.5
-^#^	MjTX-II/FA	178	32	40	11.2	19.5
-^#^	MjTX-II/FA^§^	179	42	33	6.2	18.7

^#^Structures from molecular dynamics (MD) simulations. *Reference structure for unbound MjTX-II and MjTX-II/FA MD simulation models. ^§^MD simulation at frame 170 ns.

### Molecular dynamics

**Figure 4:**
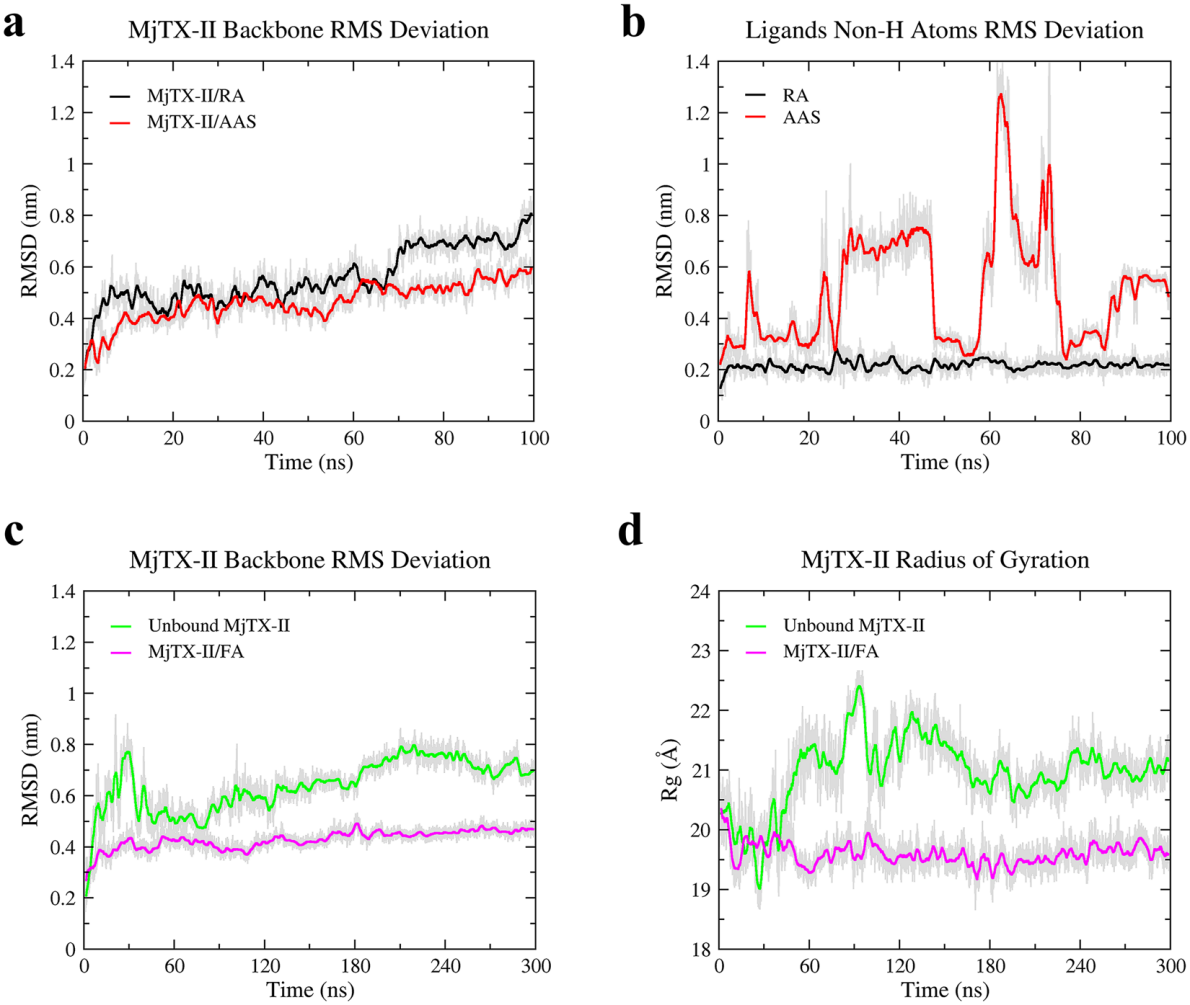
Molecular dynamics simulations with MjTX-II complexes. (**a**) Backbone atoms RMSD for MjTX-II/RA and MjTX-II/ASA complexes (black line for MjTX-II/RA and red line for MjTX-II/ASA). (**b**) Non-H atoms RMSD for RA and ASA ligands (black line - RA and red line - ASA molecules). (**c**) Backbone atoms RMSD for unbound MjTX-II (green) and MjTX-II/FA (magenta) complexes. (**d**) Radius of gyration (R_g_) for unbound MjTX-II (green) and MjTX-II/FA (magenta) complexes.

The MD simulations of the MjTX-II/RA complex resulted in average RMS deviation of frame trajectories (ftRMSD) of the MjTX-II backbone atoms of 0.56 ± 0.11 nm (Fig. 4a) with no clear indication that the protein reached a stable conformation. The average RMS deviation of frame trajectories (ftRMSD) of the RA molecule was 0.22 ± 0.03 nm, indicating that the ligand interacts with the toxin in the same region (Fig. 4b). However, the RA effect on the MjTX-II quaternary structure results in its disturbance, forcing the toxin into a distorted conformation, evidenced by the increase of its radius of gyration (R_g_) from 20.0 to 21.8 Å and COMdisp of 31.5 Å (Table 2).

In contrast, MD simulation with the MjTX-II/ASA complex showed that the ASA molecules left their initial binding sites before 2 ns of the simulation, and subsequently, both molecules dissociated from MjTX-II or interacted non-specifically during the simulation. The unstable behavior of ASA can also be observed by the high standard deviation of ftRMSD value (0.51 ± 0.23 nm) (Fig. 4b). In addition, the MjTX-II/ASA complex did not show a clear conformation stability during the simulation (Fig. 4a) and presented a ftRMSD backbone atoms of 0.46 ± 0.08 nm.

**Figure 5:**
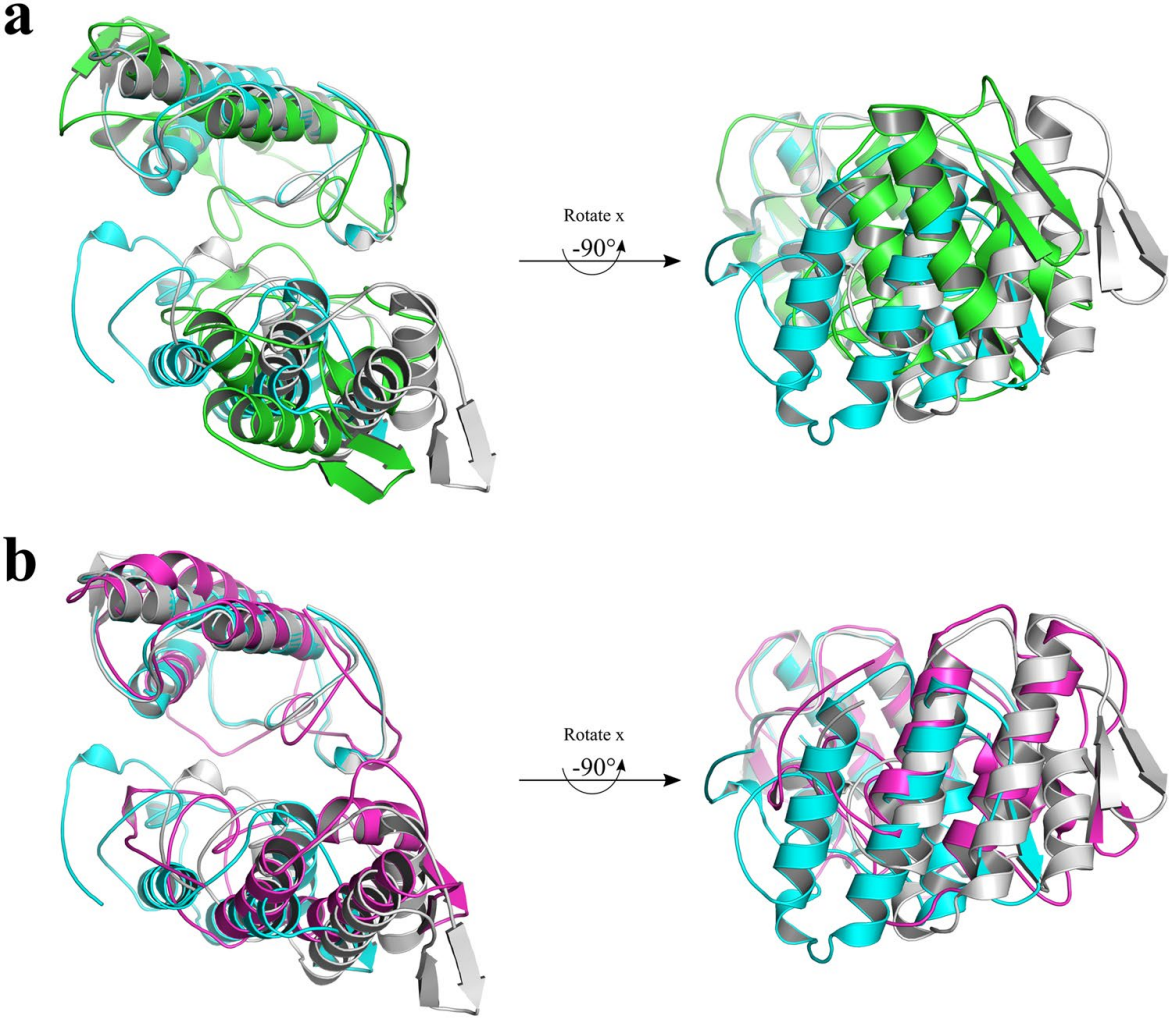
Superposition of the active MjTX-II crystal structure (MjTX-II/FA14, PDB id: 6B80) and structures from MD simulations. (**a**) Superposition between MjTX-II/FA14 crystal structure (cyan), the unbound MjTX-II model before (gray) and after (green) the MD simulation. (**b**) Superposition between MjTX-II/FA14 crystal structure (cyan), the MjTX-II/FA model before (gray) and after (magenta) the MD simulation.

In addition, other two MD simulations of 300 ns were performed aiming to test the dynamics of the distorted quaternary conformation observed in the MjTX-II/ASA complex, using the following systems: i) unbound MjTX-II and ii) unbound MjTX-II with fatty acid (FA) molecules (supposedly an activator PLA_2_-like myotoxin)^14^. FA molecules (stearic acids) were placed in the hydrophobic channel of this structure in a similar position as observed in the MjTX-II/stearic acid crystal structure (1XXS)^43^. The MD simulation of the unbound MjTX-II presented a high ftRMSD (0.64 ± 0.11 nm) (Fig. 4c) and an increase of its radius of gyration (Fig. 4d) compared with the active MjTX-II structure (MjTX-II/FA14^18^) (Table 2 and Fig. 5a). In contrast, MD simulation of the MjTX-II/FA presented more stability (ftRMSD of 0.43 ± 0.04 nm) (Fig. 4c) and the model became more globular than its initial structure (see R_g_ in Table 2 and Fig. 4d), similar to the active structure^18^.

As observed in the Table 2, different parameters (*e.g*. COMdisp and R_g_) indicated that after MD simulation, the MjTX-II/FA complex had a conformation more similar with the active form (MjTX-II/FA14) and presented a more compact structure. The Fig. 5b shows the superposition between the MjTX-II/FA structure after the simulation and the active MjTX-II/FA14 crystal structure. In the Table 2, it is also possible to observe that the MjTX-II/FA complex may reach, for example at 170 ns of the MD simulation, a even more compact conformation which its R_g_ is very similar with the active MjTX-II/FA14 crystal structure (~18 Å). In addition, at this MD simulation moment the MjTX-II/FA complex presented a COMdisp decrease from 12.7 to 6.2 Å.

## Discussion

### Functional tests and inhibition efficiency of the ligands

Similar to other PLA_2_-like proteins, MjTX-II can induce the blockage of twitches in mouse PD preparations using indirect and direct stimuli^17,20,40,41,44–48^. This paralyzing activity promoted by PLA_2_-like proteins has been proposed to result from the destabilization of the muscle membrane^38^. Initially, this membrane disarrangement results in the loss of permeability control to ions and macromolecules^49–51^. Thus, there is cellular depolarization, including the re-equilibrium of Na^+^, K^+^ and Cl^−^ ions^38,49,52^, the influx of extracellular Ca^2+^ ions^53,54^ and the release of intracellular Ca^2+^ ions^55^. Thus, an increase in the sarcoplasmic Ca^2+^ concentration promotes a series of adverse events, such as muscle contracture^49^, hypercontraction of myofilaments, mitochondrial damage and activation of proteases and Ca^2+^-dependent PLA_2_^50,51^, which amplifies the process of muscle injury^52,53^. Finally, the ATP released by the injured sarcolemma diffuses into neighboring regions of muscle fibers unaffected by the toxin, activating its P2X purinergic receptors in these muscle fibers with the consequent transmembrane passage of Na^+^, K^+^ and Ca^2+^ ions^56–59^. This ion movement results in the depolarization of these newly affected regions and an amplification of the muscle injury process in regions away from the initially disrupted sarcolemma.

Herein, we tested the ability of RA molecules to neutralize this destabilizing membrane activity through the paralyzing effect promoted by MjTX-II. Thus, we demonstrated that RA significantly reduces the blockage of indirectly evoked muscle induced by MjTX-II (Fig. 1), similarly to observed against PrTX-I, a PLA_2_-like myotoxin from *Bothrops pirajai* venom^20^. Ticli and colleagues (2005), using different technical approaches, observed the antimyotoxic potential of RA against the crude venom of *Bothrops jararacussu* and against the main PLA_2_-like myotoxins (BthTX-I and II) from this venom, as well the enhancement effect of serum therapy by this inhibitor. Furthermore, the authors exclude the proteolytic degradation of the toxin as a potential mechanism involved in the inhibition by RA^21^. Analogously, other molecules also presented an inhibitory effect against muscle paralysis promoted by PLA_2_-like toxins, such as suramin against MjTX-II^17^ and BthTX-I^41^ and Zn^2+^ ions^19^ and chicoric acid against BthTX-I^23^.

ASA is the most widely used drug in the world^60^ and, since the 1970s, it has been known that mechanism of anti-inflammatory, analgesic and antipyretic activities by this molecule occurs through cyclooxygenase (COX) inhibition^35,60^. Furthermore, other studies have investigated different applications of ASA, including its action against whole venom from *Daboia russelii* (*Viperidae*)^36^ and its toxic effects against catalytic PLA_2_^61–64^. Singh and colleagues (2005) proposed the structural basis of an elapid PLA_2_ inhibition by ASA, but no functional assays were performed^65^. Recently, ASA was evaluated against the catalytic inhibitory activity of a pancreatic PLA_2_^37^ (a secreted PLA_2_ with similar activity to PLA_2_s from *Viperidae* snake venom^66^) by bioinformatics and affinity assays. A weak interaction between ASA and the enzyme by affinity assays was observed, and a possible interaction at the Ca^2+^ binding loop was suggested by docking assays^37^. Thus, to test the ability of ASA to inhibit PLA_2_-like toxins, we performed the first tests of its interaction with a PLA_2_-like toxin and the physiological-pharmacological inhibition assays. Interestingly, despite the structural similarities between catalytic PLA_2_ and myotoxic PLA_2_-like toxins (without catalytic activity), ASA did not inhibit the blockage of indirectly evoked muscle contractions promoted by MjTX-II (Fig. 1).

### Structural evidence for MjTX-II inhibition by rosmarinic acid

**Figure 6:**
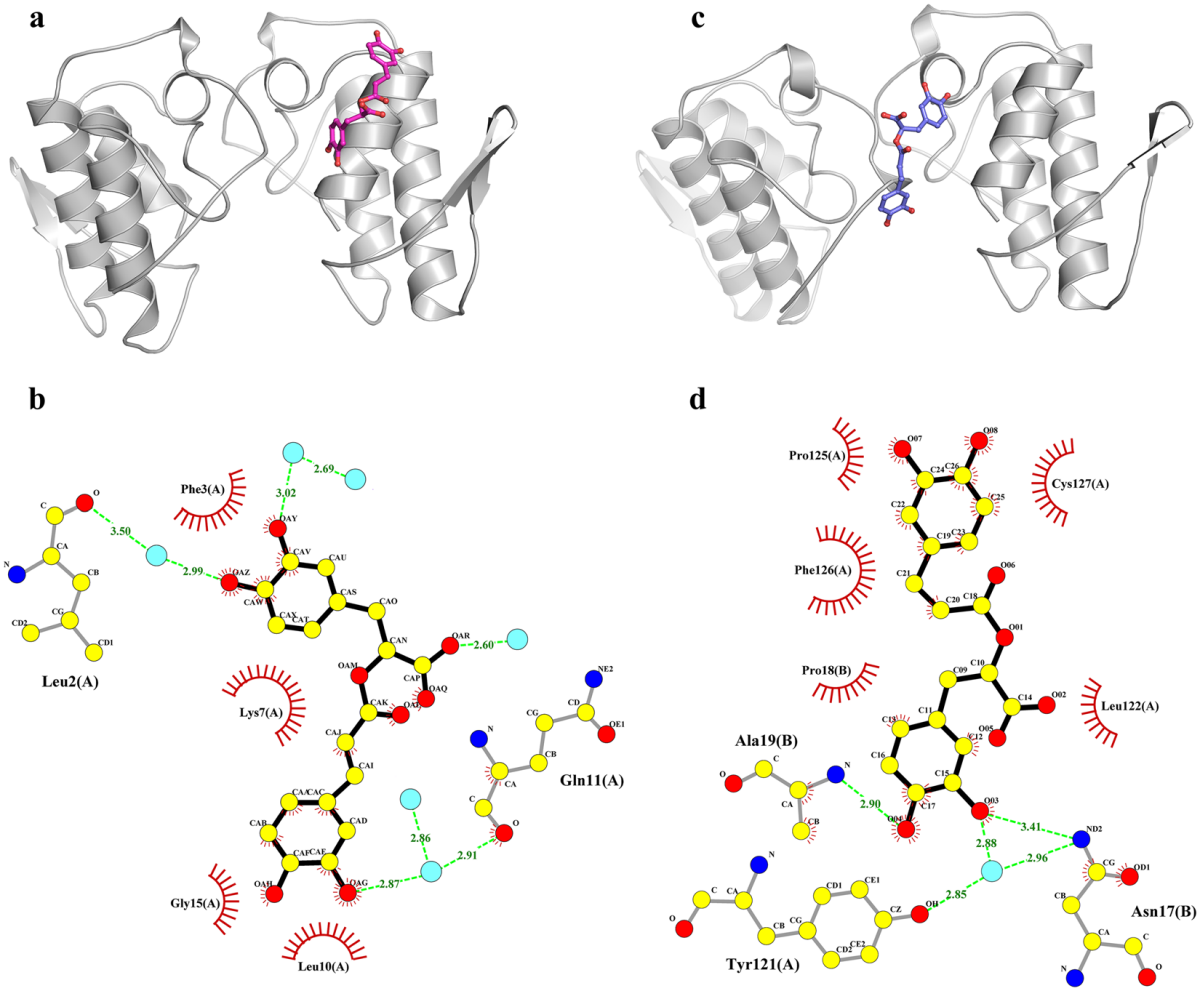
Comparison between MjTX-II/RA and PrTX-I/RA crystal structures. (**a**) Crystal structure of the PrTX-I/RA (PDB id: 3QNL) is shown as cartoon representation and RA inhibitor (magenta) is illustrated in stick representation. (**b**) Interaction of RA molecule in the PrTX-I structure. The representation of the interactions of RA was depicted as polar contacts (broken lines) and hydrophobic contacts (arcs with radiating spokes). Water molecules are showed as cyan spheres. (**c**) Crystal structure of the MjTX-II/RA is shown as cartoon representation and RA inhibitor (dark blue) is illustrated in stick representation. (**d**) Interaction of RA molecule in the MjTX-II structure. The representation of the interactions of RA was depicted as polar contacts (broken lines) and hydrophobic contacts (arcs with radiating spokes).

To date, only one crystal structure of a PLA_2_-like/RA complex, PrTX-I/RA (PrTX-I from *B. pirajai* venom complexed to RA), has been deposited in the Protein Data Bank. In this structure, an inhibitor molecule interacts with the N-terminal residues of a toxin monomer^20^ (Fig. 6a,b), and therefore, the RA molecule physically blocks the access of fatty acid molecules to the hydrophobic channels of PrTX-I. According to the current myotoxic mechanism proposed for PLA_2_-like toxins^14,15^, the entrance of a fatty acid (FA) molecule in the hydrophobic channel of the toxin is the first step of the molecular mechanism and leads to the allosteric activation of these toxins, with the subsequent exposure and alignment of their membrane docking and disruption sites (MDoS and MDiS, respectively). Thus, the RA molecule may prevent the activation of PrTX-I by blocking its hydrophobic channel.

Similar to the PrTX-I/RA complex, the crystal structure of the MjTX-II/RA complex revealed an inhibitor molecule interacting with the toxin; however, RA interacts with both monomers simultaneously and in a different region of MjTX-II (Fig. 6c). The first benzene ring region of the RA molecule (derived from the diphenyl-lactic acid portion) interacts with the N-terminus of monomer B (Asn17, Pro18 and Ala19 residues). The second benzene ring region of the RA molecule (derived from the caffeic acid portion) interacts with residues of the C-terminal region of monomer A (Leu122, Phe126 and Cys127 residues) (Fig. 6d), which includes the MDiS region.

Interestingly, RA and suramin^17^ are efficient inhibitors for MjTX-II and other PLA_2_-like toxins (*e*.*g*., BthTX-I, PrTX-I, MjTX-I, BaspTX-II and Ecarpholin S), but they bind to different regions of these toxins and display different inhibition mechanisms^20–22,41,67^. Furthermore, it is important to highlight that several structural studies with MjTX-II complexes^16–18,43^ demonstrated that the binding of ligands in MjTX-II is always different when compared to other PLA_2_-like toxins^20,22,39,68^. This fact was attributed to a residue insertion (Asn120) and two residue mutations (at positions 32 and 121), which cause the binding of additional ligands (*e*.*g*., fatty acids or PEG) or interactions in different regions (RA and suramin).

Several functional/structural studies with PLA_2_-like toxins and ligands (*e*.*g*., aristolochic and chicoric acids, suramin and Zn^2+^ ions)^17,19,23,39^ have demonstrated that their binding to the MDiS region is associated with the inhibition of these myotoxins, such as that observed in the crystal structure and functional data of the MjTX-II/RA complex presented here. Thus, an explanation of these phenomena is probably the lack of membrane disruption function of PLA_2_-like toxins.

### Acetylsalicylic acid binds to MjTX-II but does not inhibit its myotoxicity

The crystal structure of the MjTX-II/ASA complex reveals that an ASA molecule is bound in the hydrophobic channel of each MjTX-II monomer. Thus, these two ASA ligands establish hydrophobic interactions with several residues, some of which are conserved from catalytic PLA_2_s, such as His48, Tyr52 and Gly30, and are essential for the coordination of co-factor Ca^2+^ and for the catalytic activity of those enzymes^10,69^. Therefore, at first sight, the results may suggest that ASA inhibits PLA_2_-like toxins because its binding may prevent the activation of the toxin by fatty acid molecules (the first step of the proposed myotoxic mechanism). However, in the same functional experiments with PD preparations, as performed by myographic assays with MjTX-II/RA treatment, the inhibitory ability of ASA against the neuromuscular blockage induced by MjTX-II was not observed.

**Figure 7:**
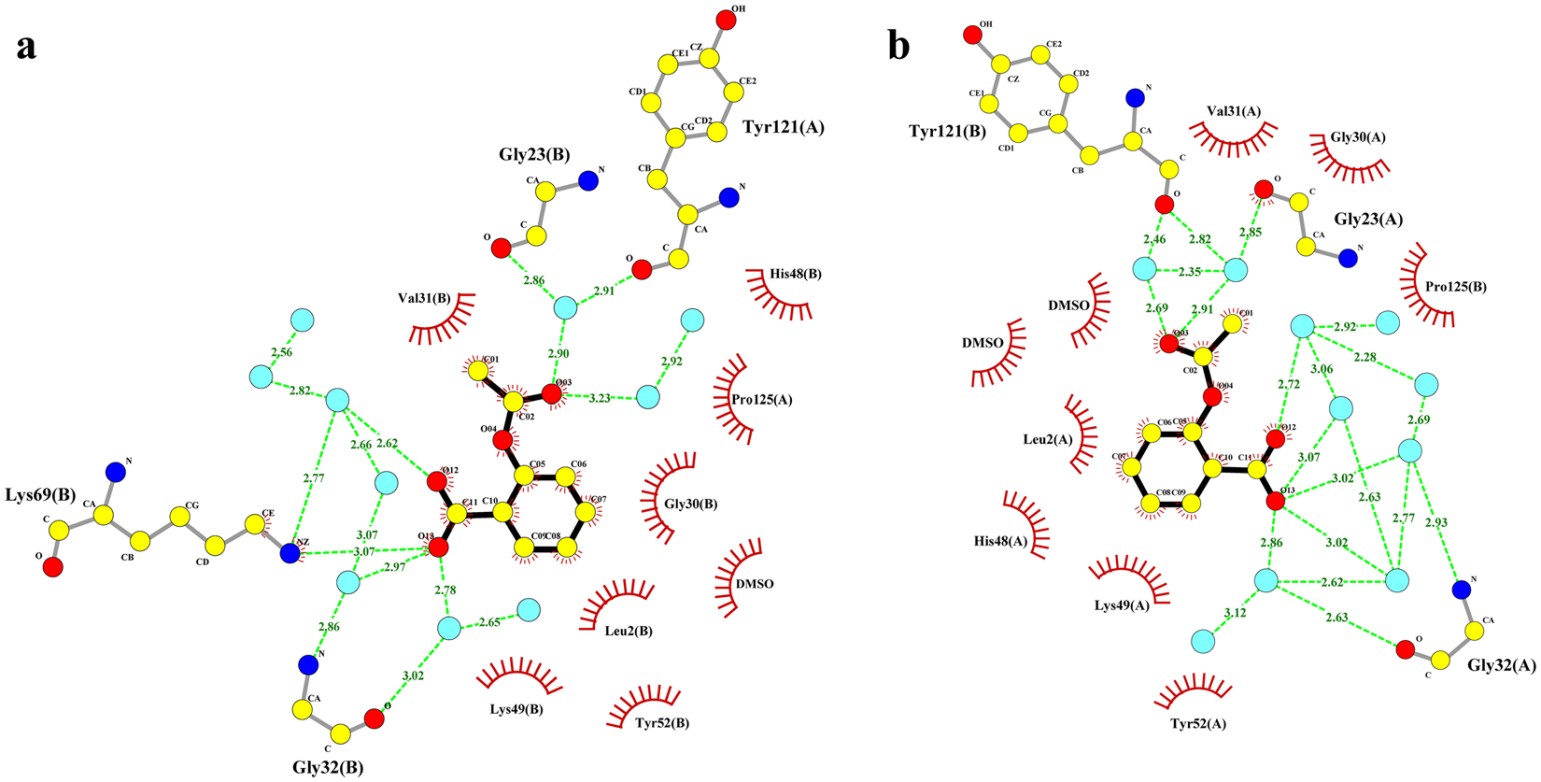
Interaction of ASA molecules with MjTX-II. ASA molecule interacting with hydrophobic channel residues from MjTX-II - monomer A (**a**) and monomer B (**b**). The representation of the interactions was depicted as polar contacts (broken lines) and hydrophobic contacts (arcs with radiating spokes). Water molecules are showed as cyan spheres.

One explanation for these contrasting observations may be related to the low stability of the ASA molecules in the complex with the toxin, as observed in MD simulations (Fig. 4b) and due to the high content of water molecules mediating the interaction between the ligand and the toxin (Fig. 7). This eventual instability may lead to its displacement by FA molecules present in the isolated organ-bath chamber, promoting a return to the active form of MjTX-II^18^.

The presence of FA molecules in the chamber may be explained by the method employed in this assay^70^. During the removal of PD preparations from animals and their assembly in these chambers, PD preparations are sufficiently manipulated to cause initial lesions of some muscle fibers, disrupting their sarcolemma (composed of phospholipids, FA molecules and several other macromolecules) with the consequent release of their intracellular content. Although the PD preparation is subjected to washing procedures after its assembly and a stabilization period of the evoked contractions occurs before the beginning of the assays, the lesions remain in the PD preparations with continuous release of the intracellular content from injured fibers, including cytosolic phospholipases and other proteases that are activated by Ca^2+^ ions^50,51^. Thus, apart from the remnants of FA molecules between the PD preparation washes, these cytosolic proteases can amplify the process of muscle injury^52,53^, releasing FA molecules prior to toxin addition into the chamber.

### MD simulations with MjTX-II/RA, MjTX-II/ASA and MjTX-II/FA shed light on the inhibition of the myotoxic mechanism

**Figure 8:**
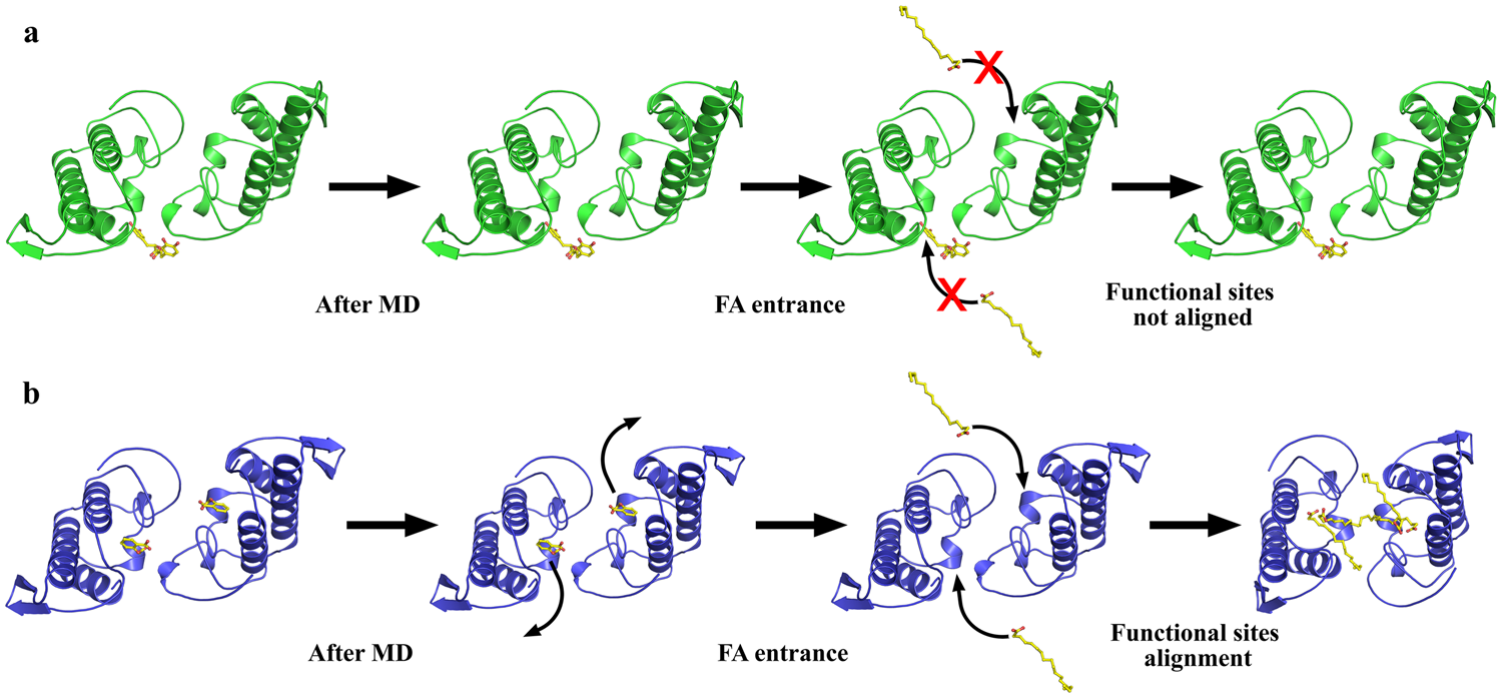
Schematic representation of structural changes caused by ligands. (**a**) In the MjTX-II/RA structure, the RA ligand is kept tight bound to the protein and blocks the interaction of fatty acids with the toxin, preventing the alignment of its functional sites. (**b**) In the MjTX-II/ASA structure, ASA ligands interact with low stability to the toxin, as observed in MD simulations, thus, fatty acids may bind with the toxin and, consequently, its functional sites may be exposed to the solvent.

Aiming to further understand the observations achieved with myographic and crystallographic methods in the present study, the dynamic of the inhibitory process was studied by MD simulations, and four different systems were considered: (*i*) MjTX-II/RA, (*ii*) MjTX-II/ASA, (*iii*) unbound MjTX-II and (*iv*) MjTX-II/FA, as described in the results section. Thus, we observed that (*i*) the RA molecule interacted with the toxin throughout the simulation and the toxin reaches a even more distorted quaternary structure (Fig. 4b); (*ii*) ASA molecules dissociated from MjTX-II at the beginning of the simulation (Fig. 4b), but the quaternary structure presented a reasonable conformational stability throughout the simulation (Fig. 4a); (*iii*) the removal of ligand molecules from the MjTX-II/ASA complex (unbound MjTX-II) was not sufficient to lead the complex to an undistorted conformation until the end of the simulation, as expected for the *apo*-MjTX-II structure^18^ (Fig. 4c,d and Table 2); and (*iv*) in the presence of FA molecules, the quaternary structure of MjTX-II (starting from an initial distorted conformational MjTX-II/ASA model) became more globular and symmetric (Fig. 8).

Therefore, these findings highlight the efficiency of RA as inhibitor of the toxic effects promoted by MjTX-II as observed by functional methods. The results suggest that RA inhibits this toxin not only by blocking its MDiS residues in a monomer but also by distorting the quaternary structure of MjTX-II, leading to misalignment the MDiS and MDoS regions from both monomers, which affects its myotoxic activity.

Moreover, the dissociation of ASA in the beginning of the MD simulation suggests low stability and/or affinity of the ligand in its binding site. This observation is compatible with functional assays that did not detect myotoxicity reduction or neutralization in its presence, as discussed in the previous sections.

FA molecules have an important role for oligomeric changes that lead to the activation of the myotoxic mechanism proposed for PLA_2_-like toxins^14,15^, but in the case of MjTX-II, their binding is not necessary for the alignment of the functional sites of this particular toxin^18^. However, the MD simulations performed here showed that the distorted starting model with no ligands (unbound MjTX-II) was not able to assume the active state (symmetric conformation), which may only obtain after the addition of FA molecules. These findings reinforce previous structural data with MjTX-II that showed that while FA molecules do not lead to important quaternary conformation changes, their binding has a direct influence on some side-chain orientations in the dimeric interface^18^, leading to stabilization of the dimeric conformation of MjTX-II.

## Conclusions

In the present study, functional, crystallographic and bioinformatics assays involving MjTX-II, a PLA_2_-like toxin with structural particularities, and two potential inhibitors were performed. RA, previously tested as an efficient inhibitor for a PLA_2_-like toxin (PrTX-I), was demonstrated to be highly efficient for the inhibition of MjTX-II in mouse PD preparations. Interestingly, despite similar inhibitory effects against MjTX-II and PrTX-I, the RA binding sites in these toxins are different. In the case of MjTX-II, the inhibitory effect is attributed to binding in the MDiS region, which prevents the disruptive activity of the toxin. In addition, the distorted quaternary conformation of MjTX-II after RA binding may also contribute to its inactivity. In contrast, ASA, a ligand previously tested in catalytic PLA_2_, was not able to prevent the paralyzing effect of MjTX-II, despite its binding in the MjTX-II/ASA crystal structure. The MD simulation with MjTX-II/ASA showed the low stability of this ligand in the hydrophobic channel of MjTX-II, suggesting that this molecule may be replaced with another molecule with higher affinity (such as fatty acids) in the functional assays, which can then activate the toxin. Finally, MD simulations of a MjTX-II/FA model led to a symmetric and stable structure, which reinforces the importance of fatty acids for the stabilization of the toxin. This combination of functional, structural and bioinformatics assays used here can methodologically contribute to the design of effective antiophidic molecules.

## Experimental Procedures

### Toxin isolation and ligands source

Freeze-dried crude venom (150 mg) was solubilized in 0.05 M ammonium bicarbonate pH 8.0 and subjected to ion exchange chromatography. The fraction corresponding to MjTX-II was obtained by a gradient of 0.05 to 0.5 M ammonium bicarbonate pH 8.0, as described by Soares and colleagues^71^. For contaminant removal, this fraction was subjected to reversed-phase chromatography, with a gradient of 0–66.5% acetonitrile (in 0.1% trifluoroacetic acid) in a C18 column (Shimadzu). Acetylsalicylic acid (ASA) and rosmarinic acid (RA) were purchased from Sigma-Aldrich, St. Louis, Missouri, USA.

### Functional studies

Adult male mice (25–30 g) were euthanized by exsanguination after cervical dislocation to remove the phrenic nerve-diaphragm muscle and mounted vertically (under a resting tension of 5 g) in a conventional isolated organ-bath chamber containing 15 mL of physiological solution (135 mM NaCl, 5 mM KCl, 1 mM MgCl_2_, 2 mM CaCl_2_, 15 mM NaHCO_3_, 1 mM Na_2_HPO_4_ and 11 mM glucose). This solution was constantly bubbled with carbogen (95% O_2_ and 5% CO_2_) and maintained at 35 ± 2 °C. The phrenic-diaphragm (PD) preparation was attached to an isometric force transducer (Grass-Telefactor, FT03) for recording twitch tension. The transducer signal output was amplified and recorded on a computer, via a transducer signal conditioner (Gould Systems, 13-6615-50), with the AcquireLab Data Acquisition System (Gould). Indirect contractions were evoked by rectangular pulses (0.2 Hz, 0.5 ms) and a supramaximal intensity delivered from an electronic stimulator (Grass-Telefactor, S88K) and applied to the phrenic nerve by means of a suction electrode.

Preparations were stabilized for 45 minutes before the treatment. For inhibition experiments, 1 µM MjTX-II dissolved in Ringer’s physiological solution was mixed with RA and ASA at a 1:10 and 1:20 (m/m) toxin:drug ratio, respectively. Mixtures were incubated for 30 minutes at 35 °C (± 2 °C). Control experiments were performed in the absence of toxin and in the presence of RA (10 µM) or ASA (20 µM) alone. The results are expressed as the mean ± S.E. Data were analyzed by ANOVA complemented with the Tukey-Kramer test. Values of *P* < 0.05 were considered significant.

The animals were supplied by UNESP (Biotério Central da Universidade Estadual Paulista, Botucatu, SP, Brazil). The Institutional Animal Care and Use Committee (Institute of Biosciences - São Paulo State University - UNESP) approved this study (number 033/05). The animal procedures were performed in accordance with the guidelines prepared by the Committee on the Care and Use of Laboratory Animal Resources, National Research Council, USA.

### Crystallization and X-ray data collection

The purified fraction of MjTX-II used for co-crystallization was concentrated up to 10 mg.mL^−1^ and solubilized in 20 mM ammonium bicarbonate, pH 8.0. ASA was dissolved in 100% dimethyl sulfoxide (DMSO) and RA was dissolved in 95% ammonium bicarbonate (20 mM, pH 8.0) and 5% ethanol to obtain a molar ratio of 1:8 (protein:ligand, for both compounds) in crystallization drops. Crystals from MjTX-II/RA and MjTX-II/ASA complexes were obtained by a conventional hanging drop vapor-diffusion method^72^ at constant temperature of 291 K for approximately 20 days, from a mixture of 1 µL of protein/inhibitor solution (previously incubated for 30 minutes) and 1 µL of reservoir solution and equilibrated against a reservoir (500 µL). The reservoir solution was similar to that previously found in the literature for this toxin and was composed of PEG 4000, Tris HCl pH 8.5 and lithium sulfate (MjTX-II/RA) and PEG 4000, isopropanol and sodium citrate (MjTX-II/ASA)^16–18^.

The crystals were mounted in a nylon loop and flash-cooled in a stream of liquid nitrogen using no cryoprotectant for X-ray diffraction data collection. The datasets were obtained using a synchrotron radiation source (MX2 station, Laboratório Nacional de Luz Sincrotron (LNLS), Campinas, Brazil) and a PILATUS 2 M detector (Dectris) at a wavelength of 1.458 Å (at 100 K).

### Structure determination and refinement

The dataset corresponding to MjTX-II/RA was processed using the HKL2000 v.1.8.4 software package^73^, and the MjTX-II/ASA dataset was processed using AutoPROC v.1.0.5 software^74^.

Both crystal structures were solved by the Molecular Replacement Method using the software PHASER^75^ from PHENIX software package v.1.12^76^ and the monomer A atom coordinates of MjTX-II/FA14 (PDB access code 6B80) as a search model. The modeling, ligand insertion, solvent molecules and manual refinement process were performed using Coot v.0.8.9 software^77^. Structural automated refinement of models was performed by PHENIX software package v.1.12^76^, and the structural quality was checked using PHENIX software package v.1.12 and MolProbity software^78^.

### Structural comparative analysis

For the structural comparisons, the structures of MjTX-II/RA and MjTX-II/ASA (this work), *apo*-MjTX-II (PDB id: 6B84), MjTX-II/FA8 (PDB id: 6B81), MjTX-II/FA14 (PDB id: 6B80)^18^, MjTX-II/PEG4K (PDB id: 4KF3)^16^ and PrTX-I/RA (Piratoxin-I from *Bothrops pirajai* - PDB id: 3QNL)^20^ were used. Molecular comparison of the structures was performed using Coot v.0.8.9 software^77^. All structural figures was generated using PyMOL v.1.3 software^79^ and LigPlot^+^ v.1.4.5^80^.

In order to analyze the conformations, the center of mass (COM) displacement measure was calculated. This measurement considers the active MjTX-II structure (MjTX-II /FA14, PDB id: 6B80) as reference. For each subset the structures (Table 2), both monomers A were superposed (C_α_ atoms) and COM was calculated for each monomers B; subsequently, the COM distance between both monomers B was calculated by Euclidean distance, resulting in a displacement value called COMdisp.

### Molecular dynamics (MD) simulation

All MD simulations were performed using GROMACS (Groningen Machine for Chemical Simulation) v.5.0.5^81^ under GROMOS96 54a7 force field^82^. MjTX-II protonation states were set to pH 8.0 using PROPKA3 server^83^, and each complex was placed in a triclinic box. The systems were solvated using simple point charge (SPC) water models to maintain the crystallographic water molecule positions and were equilibrated with 100 mM NaCl. A minimization step was applied using the Steepest Descent algorithm to reach a system energy below 100 kJ/mol/nm, and then, MjTX-II, RA, ASA and FA were restrained to perform an 1 ns NVT step using a V-rescale thermostat^84^ at 310 K followed by an 1 ns NPT step adding Berendsen barostat^85^ at 1 bar to accommodate the systems. Furthermore, unrestrained MD simulations were performed for each system using a Nose-Hoover thermostat^86,87^ and Parrinello-Rahman barostat^88^.

MD simulations of 100 ns were performed using the MjTX-II/ASA and MjTX-II/RA crystallographic structures. MD simulations of 300 ns were performed using MjTX-II/ASA crystal structure, only removing ASA molecules (unbound MjTX-II system), or removing ASA molecules and adding stearic acid molecules in each hydrophobic channel (MjTX-II/FA system). Stearic acid molecules were added manually, based on the crystallographic structure of MjTX-II complexed with stearic acid (PDB id: 1XXS)^43^ using Coot v.0.8.9 software^77^.

RA, ASA and FA topologies were built using the online server Automated Topology Builder (ATB) v.2.2 (https://atb.uq.edu.au/)^89^, which uses Quantum Mechanics calculations to determine bonded and non-bonded information, while charges were manually corrected to harmonize with force field parameters.

## References

[1] Otero R (2002). Complications of *Bothrops*, *Porthidium*, and *Bothriechis* snakebites in Colombia. A clinical and epidemiological study of 39 cases attended in a university hospital. Toxicon.

[2] Warrell DA (2010). Snake bite. Lancet.

[3] Gutierrez JM, Theakston RD, Warrell DA (2006). Confronting the neglected problem of snake bite envenoming: the need for a global partnership. PLoS Med.

[4] Kasturiratne A (2008). The global burden of snakebite: a literature analysis and modelling based on regional estimates of envenoming and deaths. PLoS Med.

[5] Gutierrez JM (1998). Neutralization of local tissue damage induced by *Bothrops asper* (terciopelo) snake venom. Toxicon.

[6] Lomonte B, Angulo Y, Calderon L (2003). An overview of lysine-49 phospholipase A_2_ myotoxins from crotalid snake venoms and their structural determinants of myotoxic action. Toxicon.

[7] Calvete JJ (2009). Snake venomics and antivenomics of *Bothrops colombiensis*, a medically important pitviper of the *Bothrops atrox-asper* complex endemic to Venezuela: Contributing to its taxonomy and snakebite management. J Proteomics.

[8] Ghazaryan NA (2015). Phospholipases A_2_ from Viperidae snakes: Differences in membranotropic activity between enzymatically active toxin and its inactive isoforms. Biochim Biophys Acta.

[9] Gutierrez JM, Lomonte B (2013). Phospholipases A_2_: unveiling the secrets of a functionally versatile group of snake venom toxins. Toxicon.

[10] Arni RK, Ward RJ (1996). Phospholipase A_2_ - a structural review. Toxicon.

[11] dos Santos JI, Soares AM, Fontes MR (2009). Comparative structural studies on Lys49-phospholipases A_2_ from *Bothrops* genus reveal their myotoxic site. J Struct Biol.

[12] Kini RM (2003). Excitement ahead: structure, function and mechanism of snake venom phospholipase A_2_ enzymes. Toxicon.

[13] Fernandes CA (2013). Structural bases for a complete myotoxic mechanism: crystal structures of two non-catalytic phospholipases A_2_-like from *Bothrops brazili* venom. Biochim Biophys Acta.

[14] Fernandes CA, Borges RJ, Lomonte B, Fontes MR (2014). A structure-based proposal for a comprehensive myotoxic mechanism of phospholipase A_2_-like proteins from viperid snake venoms. Biochim Biophys Acta.

[15] Borges RJ, Lemke N, Fontes MRM (2017). PLA_2_-like proteins myotoxic mechanism: a dynamic model description. Sci Rep.

[16] Salvador GH (2013). Structural and functional studies with mytoxin II from *Bothrops moojeni* reveal remarkable similarities and differences compared to other catalytically inactive phospholipases A_2_-like. Toxicon.

[17] Salvador GH (2015). Structural and functional evidence for membrane docking and disruption sites on phospholipase A_2_-like proteins revealed by complexation with the inhibitor suramin. Acta Crystallogr D Biol Crystallogr.

[18] Salvador GHM, Dos Santos JI, Borges RJ, Fontes MRM (2018). Structural evidence for a fatty acid-independent myotoxic mechanism for a phospholipase A_2_-like toxin. Biochim Biophys Acta.

[19] Borges RJ (2017). Functional and structural studies of a Phospholipase A_2_-like protein complexed to zinc ions: Insights on its myotoxicity and inhibition mechanism. Biochim Biophys Acta.

[20] Dos Santos JI (2011). Structural and functional studies of a bothropic myotoxin complexed to rosmarinic acid: new insights into Lys49-PLA_2_ inhibition. PLoS One.

[21] Ticli FK (2005). Rosmarinic acid, a new snake venom phospholipase A_2_ inhibitor from *Cordia verbenacea* (Boraginaceae): antiserum action potentiation and molecular interaction. Toxicon.

[22] Murakami MT (2005). Inhibition of myotoxic activity of *Bothrops asper* myotoxin II by the anti-trypanosomal drug suramin. J Mol Biol.

[23] Cardoso FF (2018). Structural basis of phospholipase A_2_-like myotoxin inhibition by chicoric acid, a novel potent inhibitor of ophidian toxins. Biochim Biophys Acta Gen Subj.

[24] Mendes MM (2013). Triacontyl *p-*coumarate: an inhibitor of snake venom metalloproteinases. Phytochemistry.

[25] Baraldi PT (2016). A novel synthetic quinolinone inhibitor presents proteolytic and hemorrhagic inhibitory activities against snake venom metalloproteases. Biochimie.

[26] Soares AM (2005). Medicinal plants with inhibitory properties against snake venoms. Curr Med Chem.

[27] Marcussi S (2007). Snake venom phospholipase A_2_ inhibitors: medicinal chemistry and therapeutic potential. Curr Top Med Chem.

[28] Carvalho BM (2013). Snake venom PLA_2_s inhibitors isolated from Brazilian plants: synthetic and natural molecules. Biomed Res Int.

[29] Hage-Melim LI, Sampaio SV, Taft CA, Silva CH (2013). Phospholipase A_2_ inhibitors isolated from medicinal plants: alternative treatment against snakebites. Mini Rev Med Chem.

[30] Guimaraes CL (2014). Biodiversity as a source of bioactive compounds against snakebites. Curr Med Chem.

[31] Aung HT, Furukawa T, Nikai T, Niwa M, Takaya Y (2011). Contribution of cinnamic acid analogues in rosmarinic acid to inhibition of snake venom induced hemorrhage. Bioorg Med Chem.

[32] Petersen M, Simmonds MS (2003). Rosmarinic acid. Phytochemistry.

[33] Aung HT, Nikai T, Niwa M, Takaya Y (2010). Rosmarinic acid in *Argusia argentea* inhibits snake venom-induced hemorrhage. J Nat Med.

[34] Aung HT (2010). Biological and pathological studies of rosmarinic acid as an inhibitor of hemorrhagic *Trimeresurus flavoviridis* (habu) venom. Toxins (Basel).

[35] Vane JR (1971). Inhibition of prostaglandin synthesis as a mechanism of action for aspirin-like drugs. Nat New Biol.

[36] Wu RC, Chou PT, Chen LK (2016). Aspirin plus tirofiban inhibit the thrombosis induced by Russell’s viper venom. Thromb J.

[37] Dileep KV (2015). Comparative studies on the inhibitory activities of selected benzoic acid derivatives against secretory phospholipase A_2_, a key enzyme involved in the inflammatory pathway. Mol Biosyst.

[38] Gallacci M, Cavalcante WL (2010). Understanding the *in vitro* neuromuscular activity of snake venom Lys49 phospholipase A_2_ homologues. Toxicon.

[39] Fernandes CA (2015). Structural Basis for the Inhibition of a Phospholipase A_2_-Like Toxin by Caffeic and Aristolochic Acids. PLoS One.

[40] Cavalcante WL (2007). Neutralization of snake venom phospholipase A_2_ toxins by aqueous extract of *Casearia sylvestris* (Flacourtiaceae) in mouse neuromuscular preparation. J Ethnopharmacol.

[41] de Oliveira M (2003). Antagonism of myotoxic and paralyzing activities of bothropstoxin-I by suramin. Toxicon.

[42] Magro AJ, Fernandes CA, dos Santos JI, Fontes MR (2009). Influence of quaternary conformation on the biological activities of the Asp49-phospholipases A_2_s from snake venoms. Protein Pept Lett.

[43] Watanabe L, Soares AM, Ward RJ, Fontes MR, Arni RK (2005). Structural insights for fatty acid binding in a Lys49-phospholipase A_2_: crystal structure of myotoxin II from *Bothrops moojeni* complexed with stearic acid. Biochimie.

[44] Gallacci M, Oliveira M, Dal Pai-Silva M, Cavalcante WL, Spencer PJ (2006). Paralyzing and myotoxic effects of a recombinant bothropstoxin-I (BthTX-I) on mouse neuromuscular preparations. Exp Toxicol Pathol.

[45] Heluany NF, Homsi-Brandeburgo MI, Giglio JR, Prado-Franceschi J, Rodrigues-Simioni L (1992). Effects induced by bothropstoxin, a component from *Bothrops jararacussu* snake venom, on mouse and chick muscle preparations. Toxicon.

[46] Oshima-Franco Y (2004). The presynaptic activity of bothropstoxin-I, a myotoxin from *Bothrops jararacussu* snake venom. Basic Clin Pharmacol Toxicol.

[47] Ponce-Soto LA (2009). Neuromuscular activity of BaTX, a presynaptic basic PLA_2_ isolated from *Bothrops alternatus* snake venom. Comparative Biochemistry and Physiology C-Toxicology & Pharmacology.

[48] Stabeli RG (2006). *Bothrops moojeni* myotoxin-II, a Lys49-phospholipase A_2_ homologue: an example of function versatility of snake venom proteins. Comp Biochem Physiol C Toxicol Pharmacol.

[49] Rodrigues-Simioni L, Borgese N, Ceccarelli B (1983). The effects of *Bothrops jararacussu* venom and its components on frog nerve-muscle preparation. Neuroscience.

[50] Gutierrez JM, Lomonte B (1995). Phospholipase A_2_ myotoxins from *Bothrops* snake venoms. Toxicon.

[51] Ownby CL, Selistre de Araujo HS, White SP, Fletcher JE (1999). Lysine 49 phospholipase A_2_ proteins. Toxicon.

[52] Montecucco C, Gutierrez JM, Lomonte B (2008). Cellular pathology induced by snake venom phospholipase A_2_ myotoxins and neurotoxins: common aspects of their mechanisms of action. Cell Mol Life Sci.

[53] Gutierrez JM, Ownby CL (2003). Skeletal muscle degeneration induced by venom phospholipases A_2_: insights into the mechanisms of local and systemic myotoxicity. Toxicon.

[54] Villalobos JC, Mora R, Lomonte B, Gutierrez JM, Angulo Y (2007). Cytotoxicity induced in myotubes by a Lys49 phospholipase A_2_ homologue from the venom of the snake *Bothrops asper*: evidence of rapid plasma membrane damage and a dual role for extracellular calcium. Toxicol In Vitro.

[55] Johnson EK, Ownby CL (1994). The role of extracellular ions in the pathogenesis of myonecrosis induced by a myotoxin isolated from Broad-Banded copperhead (*Agkistrodon contortrix laticinctus*) venom. Comp Biochem Physiol Pharmacol Toxicol Endocrinol.

[56] Burnstock G (2007). Physiology and pathophysiology of purinergic neurotransmission. Physiol Rev.

[57] Di Virgilio F (2007). Liaisons dangereuses: P2X_7_ and the inflammasome. Trends Pharmacol Sci.

[58] Abbracchio MP, Burnstock G, Verkhratsky A, Zimmermann H (2009). Purinergic signalling in the nervous system: an overview. Trends Neurosci.

[59] Cintra-Francischinelli M (2010). *Bothrops* snake myotoxins induce a large efflux of ATP and potassium with spreading of cell damage and pain. Proc Natl Acad Sci USA.

[60] Vane JR, Botting RM (2003). The mechanism of action of aspirin. Thromb Res.

[61] Wang JP, Teng CM (1990). Comparison of the enzymatic and edema-producing activities of two venom phospholipase A_2_ enzymes. Eur J Pharmacol.

[62] Wang JP, Teng CM (1991). Effects of anti-inflammatory drugs on rat hind-paw swelling caused by phospholipase A_2_ from *Naja naja atra* venom. Naunyn Schmiedebergs Arch Pharmacol.

[63] Wang JP, Teng CM (1992). Roles of PMN leucocytes, platelets and some mediators in rat hind-paw oedema induced by two phospholipase A_2_ enzymes from *Trimeresurus mucrosquamatus* venom. J Pharm Pharmacol.

[64] Lobo IB, Hoult JR (1994). Groups I, II and III extracellular phospholipases A_2_: selective inhibition of group II enzymes by indomethacin but not other NSAIDs. Agents Actions.

[65] Singh RK (2005). Aspirin induces its anti-inflammatory effects through its specific binding to phospholipase A_2_: crystal structure of the complex formed between phospholipase A_2_ and aspirin at 1.9 angstroms resolution. J Drug Target.

[66] Dennis EA, Cao J, Hsu YH, Magrioti V, Kokotos G (2011). Phospholipase A_2_ enzymes: physical structure, biological function, disease implication, chemical inhibition, and therapeutic intervention. Chem Rev.

[67] Zhou X (2008). Structural characterization of myotoxic ecarpholin S from *Echis carinatus* venom. Biophys J.

[68] Fernandes CA (2010). Comparison between apo and complexed structures of bothropstoxin-I reveals the role of Lys122 and Ca^2+^-binding loop region for the catalytically inactive Lys49-PLA_2_s. J Struct Biol.

[69] Murakami MT (2006). Insights into metal ion binding in phospholipases A_2_: ultra high-resolution crystal structures of an acidic phospholipase A_2_ in the Ca^2+^ free and bound states. Biochimie.

[70] Bulbring, E. Observations on the isolated phrenic nerve diaphragm preparation of the rat. 1946. *Br J Pharmacol* **120**, 3–26; discussion 21–22 (1997). 10.1111/j.1476-5381.1997.tb06771.x PMC3224265 9142393

[71] Soares AM (1998). A rapid procedure for the isolation of the Lys-49 myotoxin II from *Bothrops moojeni* (caissaca) venom: biochemical characterization, crystallization, myotoxic and edematogenic activity. Toxicon.

[72] Ducruix, A. G., R. *Crystallization of Nucleic Acids and Proteins: A Pratical Approach*. (Oxford University Press 1992).

[73] Otwinowski Z, Minor W (1997). Processing of X-ray diffraction data collected in oscillation mode. Macromolecular Crystallography, Pt A.

[74] Vonrhein C (2011). Data processing and analysis with the autoPROC toolbox. Acta Crystallogr D Biol Crystallogr.

[75] McCoy AJ (2007). Phaser crystallographic software. J Appl Crystallogr.

[76] Adams PD (2010). PHENIX: a comprehensive Python-based system for macromolecular structure solution. Acta Crystallogr D Biol Crystallogr.

[77] Emsley P, Cowtan K (2004). Coot: model-building tools for molecular graphics. Acta Crystallogr D Biol Crystallogr.

[78] Chen VB (2010). MolProbity: all-atom structure validation for macromolecular crystallography. Acta Crystallogr D Biol Crystallogr.

[79] Schrodinger, LLC. *The PyMOL Molecular Graphics System, Version1.3r1* (2010).

[80] Wallace AC, Laskowski RA, Thornton JM (1995). LIGPLOT: a program to generate schematic diagrams of protein-ligand interactions. Protein Eng.

[81] Abraham MJ (2015). GROMACS: High performance molecular simulations through multi-level parallelism from laptops to supercomputers. SoftwareX.

[82] Schmid N (2011). Definition and testing of the GROMOS force-field versions 54A7 and 54B7. Eur Biophys J.

[83] Olsson MH, Sondergaard CR, Rostkowski M, Jensen JH (2011). PROPKA3: Consistent Treatment of Internal and Surface Residues in Empirical pKa Predictions. Journal of Chemical Theory and Computation.

[84] Bussi G, Donadio D, Parrinello M (2007). Canonical sampling through velocity rescaling. Journal of Chemical Physics.

[85] Berendsen HJC, Postma JPM, Vangunsteren WF, Dinola A, Haak JR (1984). Molecular-Dynamics with Coupling to an External Bath. Journal of Chemical Physics.

[86] Nosé S (1983). A molecular dynamics method for simulations in the canonical ensemble. Molecular Physics.

[87] Hoover WG (1985). Canonical dynamics: Equilibrium phase-space distributions. Phys Rev A Gen Phys.

[88] Parrinello, M. & Rahman, A. Polymorphic transitions in single crystals: A new molecular dynamics method. *Journal of Applied Physics* **52** (1981).

[89] Koziara KB, Stroet M, Malde AK, Mark AE (2014). Testing and validation of the Automated Topology Builder (ATB) version 2.0: prediction of hydration free enthalpies. J Comput Aided Mol Des.

